# Insulin Protects Apoptotic Cardiomyocytes from Hypoxia/Reoxygenation Injury through the Sphingosine Kinase/Sphingosine 1-Phosphate Axis

**DOI:** 10.1371/journal.pone.0080644

**Published:** 2013-12-11

**Authors:** Huan Yu, Xiangxin Che, Xiaoyuan Xu, Meirong Zheng, Yong Zhao, Wei He, Jingmou Yu, Jianjun Xiong, Weidong Li

**Affiliations:** 1 College of Basic Medical Science, Jiujiang University, Jiujiang, China; 2 Key Laboratory of Jiangxi for the Systems Bio-medicine, Jiujiang, China; II Università di Napoli, Italy

## Abstract

**Objective:**

Experimental and clinical studies have shown that administration of insulin during reperfusion is cardioprotective, but the mechanisms underlying this effect are still unknown. In this study, the ability of insulin to protect apoptotic cardiomyocytes from hypoxia/reoxygenation injury using the sphingosine kinase/sphingosine 1-phosphate axis was investigated.

**Methods and Results:**

Rat cardiomyocytes were isolated and subjected to hypoxia and reoxygenation. [γ-32P] ATP was used to assess sphingosine kinase activity. Insulin was found to increase sphingosine kinase activity. Immunocytochemistry and Western blot analysis showed changes in the subcellular location of sphingosine kinase 1 from cytosol to the membrane in cardiomyocytes. Insulin caused cardiomyocytes to accumulate of S1P in a dose-dependent manner. FRET efficiency showed that insulin also transactivates the S1P_1_ receptor. TUNEL staining showed that administration of insulin during reoxygenation could to reduce the rate of reoxygenation-induced apoptosis, which is a requirement for SphK 1 activity. It also reduced the rate of activation of the S1P receptor and inhibited hypoxia/reoxygenation-induced cell death in cardiomyocytes.

**Conclusion:**

The sphingosine kinase 1/sphingosine 1-phosphate/S1P receptor axis is one pathway through which insulin protects rat cardiomyocytes from apoptosis induced by hypoxia/reoxygenation injury.

## Introduction

Ischemia/reperfusion (I/R) can cause serious tissue damage and myocardial dysfunction. Although early reperfusion of the ischemic myocardium is the most effective means of restoring cardiac function during acute myocardial infarction, reperfusion can also contribute to the development of myocardial cell damage [1], [2]. In cardiomyocytes, reperfusion injury begins within minutes of reperfusion. Any intervention aimed at protection from this form of injury should be applied immediately after the onset of reperfusion. Several experimental and clinical studies have shown that insulin administered at the time of reperfusion protects myocardial cells from reperfusion injury [3], [4]. Application of exogenous insulin exerts prosurvival effects in cultured cardiac myocytes subjected to hypoxia. It was also found to protect isolated hearts when administered either before ischemia or at the onset of reperfusion [5]–[8]. Insulin has been shown to decrease the rate of cell death and improve cardiac function during the reperfusion period.

A great deal of evidence has shown that apoptosis plays a key role in myocardial reperfusion injury [9], [10]. In cultured cardiac myocytes, subjected to hypoxia/reoxygenation, insulin during reoxygenation was found to reduce the number of TUNEL-positive myocardial cells [11]. Insulin also shows antiapoptotic effects in ischemia-reperfusion hearts [12].

Sphingosine 1-phosphate (S1P) is a potent inducer of proliferation and inhibitor of apoptosis [13]. S1P is a bioactive lipid present in serum. It can regulate many essential cellular processes, including cell growth and survival, cell motility and invasion, angiogenesis, immune regulation, and lymphocyte trafficking. S1P is synthesized from sphingosine through a phosphorylation reaction catalyzed by the sphingosine kinases (SphKs) SphK1 and SphK2, which are highly conserved and activated by numerous stimuli. S1P exerts its functions either as a second messenger or as a ligand of five specific G-protein coupled to receptors, which are called S1P receptors (S1PR). S1PR are differentially targeted to one or multiple G-proteins. They can activate a variety of signaling pathways that cause distinct and even contrasting final cellular effects.

Activation of sphingosine kinase/sphingosine 1-phosphate mediated signaling has been identified as a critical cardioprotective pathway in acute ischemia/reperfusion injury. The use of exogenous S1P exerts prosurvival effects in cultured cardiac myocytes subjected to hypoxia and in isolated hearts treated either before ischemia or at the onset of reperfusion [14], [15]. Synthetic congeners of S1P mimic these responses. Gene-targeted mice null for the sphingosine kinase 1 isoform whose hearts are subjected to ischemia/reperfusion injury exhibit increased infarct size and respond poorly to both ischemic preconditioning and ischemic postconditioning [16], [17].

Because of the fundamental role of insulin in cardiomyocytes and its incompletely defined mechanism of action, the present work focused on the possible involvement of the SK/S1P axis in the biological effects of insulin. Data reported here demonstrate for the first time that insulin activates the activity and translocation of SphK1 in cardiomyocytes. S1P1 was here found to be transactivated by insulin. The engagement of insulin protected cardiomyocytes from apoptosis induced by hypoxia/reoxygenation via a mechanism dependent on SK activation and on the S1P1 receptor, which indicates that the SK/S1P axis plays a role in the control of the biological outcome of insulin in cardiomyocytes.

## Materials and Methods

### 1.1. Reagents

Sphingosine 1-phosphate and [γ-32P] ATP (3000 Ci/mmol) were obtained from GE Healthcare Europe (Milan, Italy). VPC23019 was obtained from Avanti Polar Lipids, Inc (Alabaster, AL, U.S.). S1P and dimethylsphingosine were obtained from Biomol (Plymouth Meeting, PA, U.S.). Sphingosine kinase inhibitor 2-(p-hydroxyanilino)-4-(p-chlorophenyl) thiazole (HACPT) was obtained from Calbiochem (La Jolla, CA, U.S.).

### 1.2. Cell cultures

All animals used in this study were handled in compliance with the International Guiding Principles for Animal Research and all procedures were approved by the University of Jiujiang Animal Care and Use Committee (permit number: 2011-0007). Cultured rat cardiomyocytes were prepared from the ventricular tissue of 2-to-3-day-old Sprague-Dawley rats, as described previously [18]. Cells were incubated overnight at 37°C under 5% (v/v) CO_2_ and 95% O_2_ (v/v) for 24 h.

### 1.3. Plasmid construction

GFP-rat SphK1 and S1P_1_-CFP, YFP-β-arrestin were constructed as described previously [19].

### 1.4. siRNA

Small interfering RNAs (siRNAs) for rSphK1 (5′-GGGCAAGGCUCUGAAGCUCdTdT-3′ and 5′-GAGCUUCAGAGCCUUGCCCdTdT-3′; dT is deoxyribosylthymine throughout) for the control siRNA (5′-UUCUCCGAACGUGUCACGUdTdT-3′ and 5′-ACGUGACACGUUCGGAGAAdTdT-3′) were synthesized. Cardiomyocytes were transfected with both siRNAs for 2 days before the assays. The transfection efficiency of the siRNAs in cells was determined using a commercially available kit (Block-iT Alexa Fluor Red Fluorescent Oligo; Invitrogen).

### 1.5. Assay of sphingosine kinase activity [20]

The assay for measurement of the production of (32)P-labeled S1P after the addition of exogenous sphingosine and [γ(32)P] adenosine-5′-triphosphate. The S1P product was purified using Bligh-Dyer solvent extraction and separated with thin-layer chromatography (TLC), and the radiolabelled S1P was quantified by exposing the TLC plate to a storage phosphor screen.

### 1.6. S1P determination

Cardiomyocytes were plated in 24-well culture plates. To each well, 160 µl of assay mixture (100 mM Tris-HCl (pH 7.4), 150 mM NaCl, 10 mM MgCl_2_, 10 mM NaF, 1 mM Na_3_VO_4_, 0.5 mM 4-deoxypyridoxine), 20 µl of 1 mM sphingosine conjugated with 0.2% fatty acid-free bovine serum albumin, 10 µl of ATP solution (20 mM ATP with [γ-^32^P] ATP (1 µCi/well)), and 10 µl of different concentrations of insulin solution were added. Cells were incubated at 37°C for 30 min. The radio-labeled S1P was extracted and separated using thin-layer chromatography [21]. The radioactivity of S1P was quantitated using a Fujix Bio-imaging Analyzer BAS 2000 (Fuji Photo Film, Japan).

### 1.7. FRET

Primary cardiomyocytes were cotransfected with a donor, S1P_1_-CFP, and an acceptor, YFP-β-arrestin, at a donor/acceptor ratio of 1∶1. After excitation at 458 nm, CFP and YFP emission spectra were collected (from cells expressing S1P1-CFP or YFP-β-arrestin alone) in eight channels, each 20 nm wide, ranging from 473 to 633 nm. Spectra were collected using the lambda mode of the Zeiss LSM 510 META confocal microscope and analytical software. Two days after cotransfection, cardiomyocytes were treated with various agonists. Each part of the membrane was subjected to fluorescence resonance energy transfer (FRET) analysis. FRET efficiency was measured after acceptor photobleaching, as described [22]. FRET efficiency (*E*) was determined using the relative fluorescence intensity of the energy donor (CFP) before (*I*pre) and after (*I*post) photobleaching of the energy acceptor (YFP): *E* = 1—(*I*pre/*I*post) [23].

### 1.8. Hypoxia-reoxygenation

All cells were changed to serum-free medium without glucose. For hypoxia-reoxygenation experiments, cells were exposed to 99% N_2_, 1% CO_2_ for 4 h using preequilibrated glucose-free media in a hypoxia chamber. Cells were then reoxygenated with media containing glucose and maintained under normoxic conditions for 20 h. Control cells were treated similarly, except they remained under normoxic conditions throughout the entire experiment.

### 1.9. TUNEL staining

Cells were stained with a commercially available TUNEL kit in accordance with the manufacturer's instructions (Roche Diagnostics). Nuclei were counter-stained with DAPI. The relative number of TUNEL-positive nuclei was calculated as sum of all double-positive nuclei divided by the sum of all nuclei per high-power field.

### 2.0. Statistical analysis

Data are expressed as means± S.E. and were compared using one-way and two-way ANOVA, followed by the Student's t test. *P*<0.05 was considered significant.

## Results

### 2.1. Effects of insulin on sphingosine kinase activity and subcellular location in cardiomyocytes

**Figure 1:**
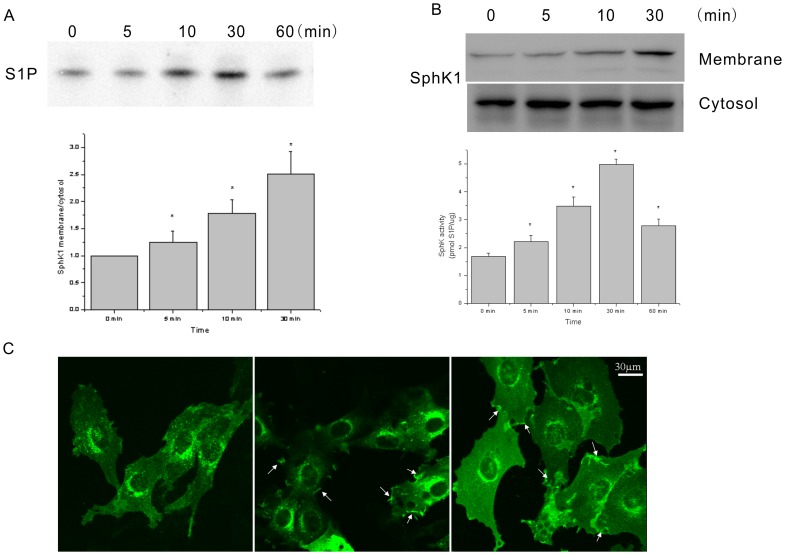
Effects of insulin on sphingosine kinase activity and subcellular localization. Serum-starved cardiomyocytes were incubated with 10 mU/L insulin for the indicated periods of time. A: Aliquots of cell extracts (40 µg) were used to assess sphingosine kinase (SphK) activity. Data represent the mean ± SEM of at least three independent experiments, each performed at least in duplicate. The difference between the effects of insulin in challenged and unchallenged cells was found to be statistically significant using Student's t tests (*P<0.05). B: Western blot analyses of SphK1 were performed in membrane and cytosolic fractions. Blots representative of at least three independent experiments are shown. The histograms represent densitometric analysis of three independent experiments. Data reported are expressed as fold increase of the membrane∶cytosol ratio. The insulin-induced increase in the SphK1 content of the membrane was found to be statistically significant by Student's t-test (*P<0.05). C: Cardiomyocytes were transfected with GFP-SphK1 for 24 h, stimulated with insulin, then observed SphK1 under confocal fluorescence microscopy. C1: cardiomyocytes transfected with GFP-SphK1 for 24 h and with 0 mU/L insulin for 10 min; C2: cardiomyocytes transfected with GFP-SphK1 for 24 h and with 5 mU/L insulin for 10 min; C3: cardiomyocytes transfected with GFP-SphK1 for 24 h and with 10 mU/L insulin for 10 min. Arrows point to membrane ruffling.

To determine whether the SK/S1P axis is involved in insulin biological action, it was first determined whether insulin was capable of regulating SK activity in cardiomyocytes. As shown in Figure 1A, data indicated that 10 mU/L insulin stimulated SphK activity. Insulin increased SphK activity in a time-dependent manner. In addition to regulating SphK activity, insulin was also responsible for rapid, transient translocation of SphK1 to the membrane fraction. This effect was appreciable within 5 minutes of incubation (Figure 1B). Overexpression of GFP-SphK1 was detected, and GFP- SphK1 was translocated to the membrane after treatment with insulin (Figure 1C). No changes in SphK protein and the location of SphK 2 were observed were observed (data not shown).

### 2.2. Dose dependence of insulin on S1P generation

**Figure 2:**
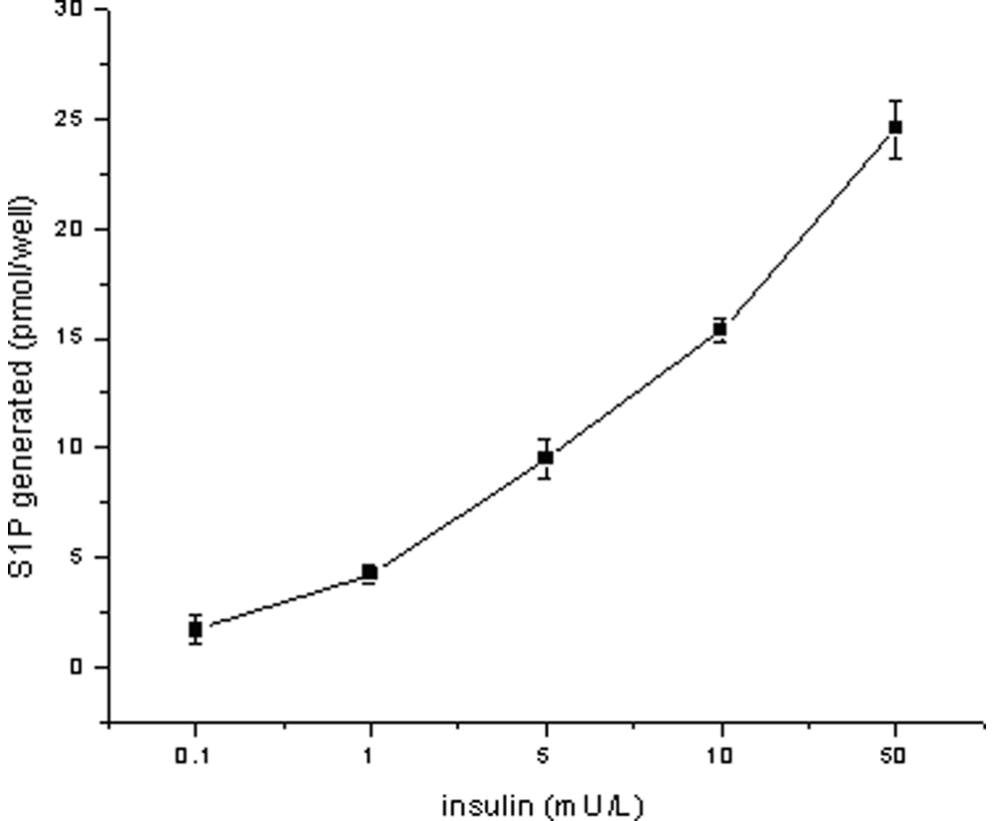
Dose dependence of the effects of insulin on S1P generation. Cardiomyocytes were incubated for 30[γ-32P] ATP. After lipid extraction, the radioactive S1P that had been generated was separated using thin-layer chromatography and quantitated. Data are means ± S.E. of three independent experiments carried out in triplicate.

Insulin increases sphingosine kinase activity and subcellular localization in cardiomyocytes. The SphK product S1P suggests that S1P may be generated after exogenous addition of insulin (Figure 2). To confirm this, insulin-induced S1P generation was measured in cardiomyocytes. As expected, exogenously added insulin caused intracellular accumulation of S1P in a dose-dependent manner. These results strongly suggest that SphK-catalyzed formation of intracellular S1P may be important to the effects of insulin on cardiomyocytes.

### 2.3. Activation of S1P receptor by insulin

**Figure 3:**
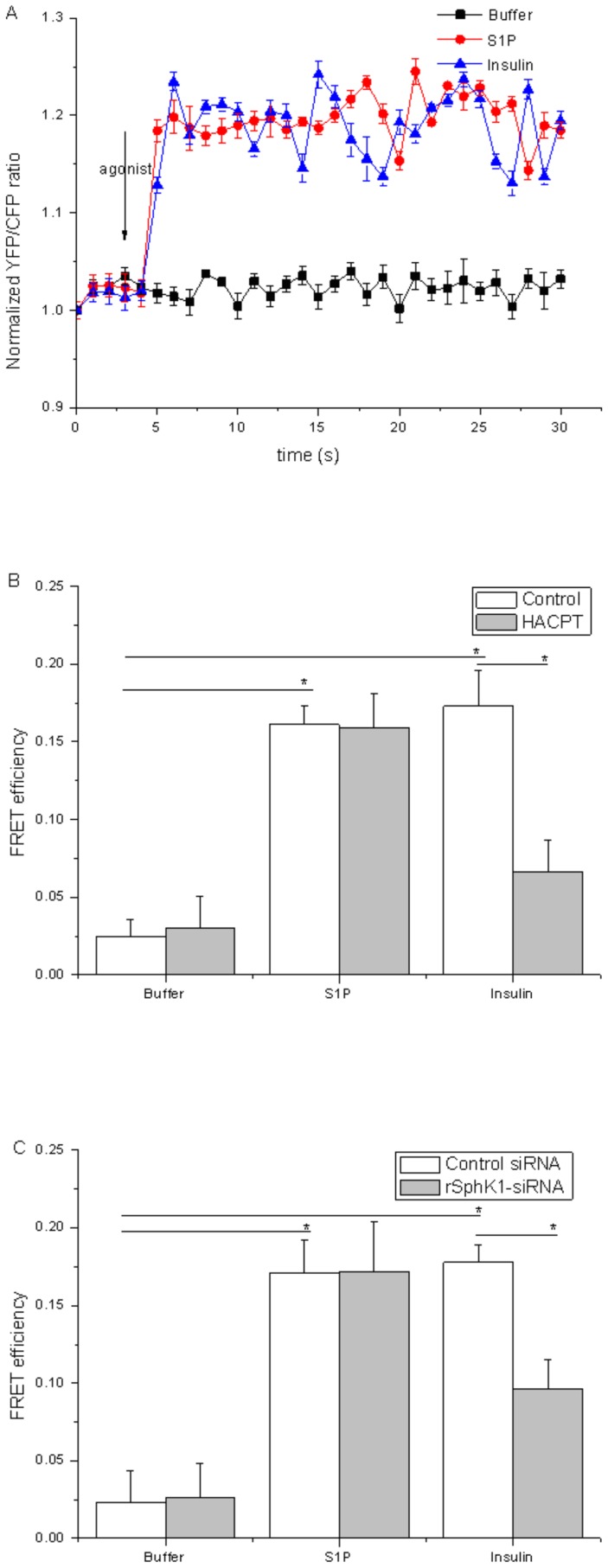
Insulin-induced S1P receptor activation as demonstrated by FRET analysis. A: Cardiomyocytes transfected with expression plasmids encoding S1P1-CFP and YFP-β-arrestin were treated either with stimuli (buffer vehicle or 500 nM S1P) or with 10 mU/L insulin and were analyzed for FRET in living cells. A representative emission ratio of the two fluorophores (excited at 458 nm) from five independent experiments is shown. B: Cardiomyocytes cotransfected with expression plasmids encoding S1P1-CFP and YFP-β-arrestin plasmids were treated with or without 50 µM HACPT for 30 min and then treated with either empty buffer, 500 nM S1P, or 10 mU/L insulin. They were then analyzed for FRET in living cells. Emissions detected from an increase in donor fluorescence after acceptor photobleaching of puncta of membrane were measured and expressed as FRET efficiency. Insulin and S1P treatment both caused a significant increase in FRET efficiencies (n = 50; a representative experiment of four independent experiments is shown; *P*<0.05, Student's paired t test). C: In some experiments, cardiomyocytes were transfected with control or rSK1-siRNAs along with plasmids encoding the fluorophore-conjugated proteins. The areas of membrane were photobleached. Then the cardiomyocytes were stimulated with either empty (buffer), 500 nM S1P, or 10 mU/L insulin, fixed, and measured for FRET efficiency. Data are expressed as means and standard errors of the means of three independent experiments carried out in triplicate.

Insulin enhanced SphK1 activity (Figure 1A) and translocation of SphK1 from the cytosol to the membranes (Figure 1B), and the dose-dependence of the effects of insulin on S1P generation prompted the question of whether the S1P newly generated by SphK1 was activating S1P receptors. FRET analyses were performed to assess S1P receptor activation. β-arrestin was found to bind to the G-protein-coupled receptor soon after receptor activation, and vectors were constructed to express two fusion proteins, S1P1-CFP and YFP-β-arrestin [24]. If β-arrestin binds to S1P1 and the CFP and YFP are in sufficiently close proximity, then excitation of CFP should stimulate emission from the YFP fluorophore in a FRET assay. This approach provided the first direct evidence of spatiotemporal activation of S1P receptors in cells. These constructs were transiently transfected into cardiomyocytes, and their emission profiles were examined using an excitation wavelength of 458 nm after the cells were subjected to various stimuli (Figure 3A). Upon stimulation with S1P and insulin, FRET was observed rapidly (within 1 s), and no detectable FRET was observed under unstimulated conditions, confirming that this FRET system functioned as intended. When cardiomyocytes were transfected with control plasmid vectors encoding CFP and YFP proteins, no FRET was observed after S1P stimulation (data not shown). The validity of the FRET signals produced by the two fluorophore-conjugated proteins was further confirmed through the use of FRET efficiency values based on dequenching of the CFP signal after specific photobleaching of the acceptor fluorophore YFP. FRET and stimulation of the S1P receptor were rendered significantly more efficient by insulin (Figures 3B and C), confirming the effects of insulin on S1P1 stimulation. Treatment of cardiomyocytes with either HACPT or rSK1-siRNA abrogated the insulin-induced increase in FRET efficiency but not the increase induced by S1P (Figure 3B), indicating that the activation S1P1 receptor had been activated near areas where S1P was generated.

### 2.4. Requirements for SphK activity and S1P1 for insulin inhibit ischemia-reperfusion-induced cell death in cardiomyocytes

**Figure 4:**
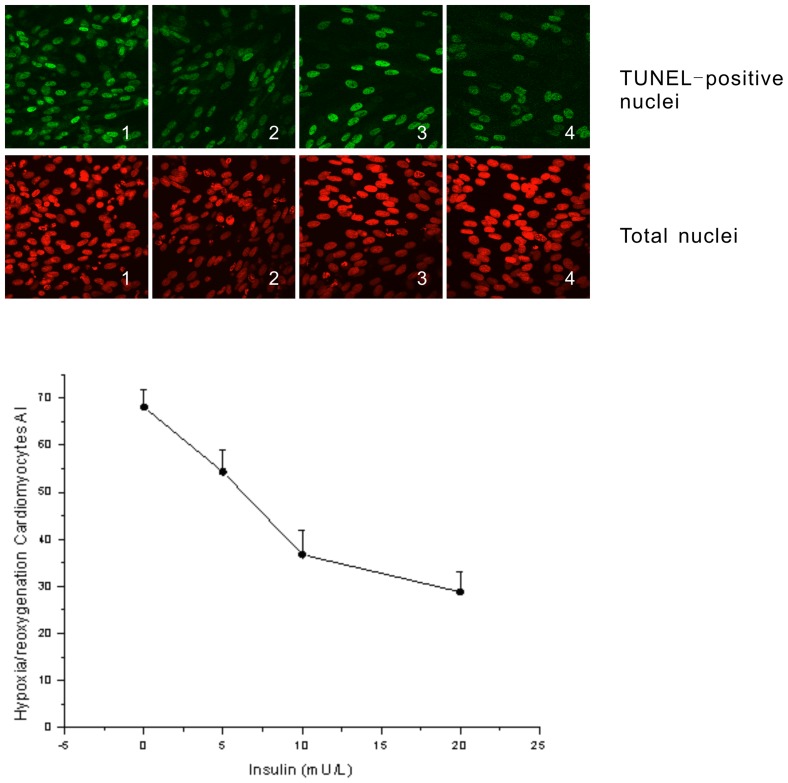
TUNEL-positive cardiomyocytes in all groups shown using confocal fluorescence microscopy. Cardiomyocytes were evaluated using hypoxia-reoxygenation. During reoxygenation, they were cultured with different concentrations of insulin. 1: 0 mU/L insulin; 2: 5 mU/L insulin; 3: 10 mU/L insulin; 4: 20 mU/L insulin. TUNEL-positive cardiomyocytes/total cardiomyocytes×100% (AI). Data are means ± S.E. of three independent experiments carried out in triplicate.

**Figure 5:**
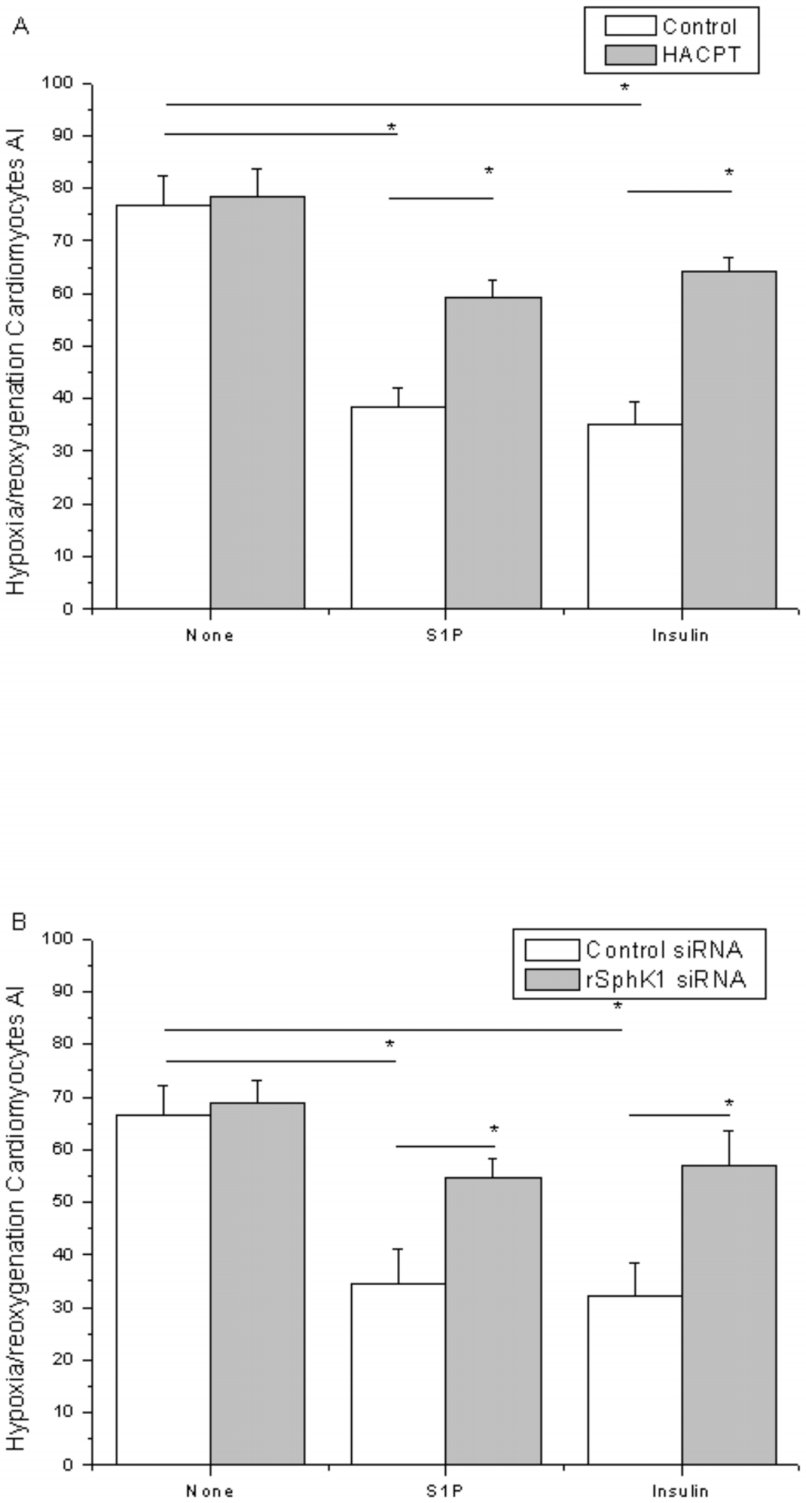
SphK activity and insulin-induced inhibition of hypoxia-reoxygenation-induced cell death in cardiomyocytes. A: Effects of HACPT on insulin in cardiomyocytes, cardiomyocytes subjected to hypoxia-reoxygenation, and cardiomyocytes during reoxygenation, all cultured with empty vehicle (vehicle, 0.05% dimethylsulfoxide and 0.05% methanol) with 50 µM HACPT, as indicated by white and gray bars, respectively. Cells were treated with control or SphK1-siRNA and cultured for 72 h. Then cells were subjected to hypoxia and treated either with nothing, S1P, or insulin. B: Cardiomyocytes were transiently transfected and reoxygenated. Cells were stimulated with nothing, S1P, or insulin during reoxygenation. TUNEL-positive cardiomyocytes/total cardiomyocytes×100% (AI). Data are means ± S.E. of five independent experiments carried out in triplicate.

**Figure 6:**
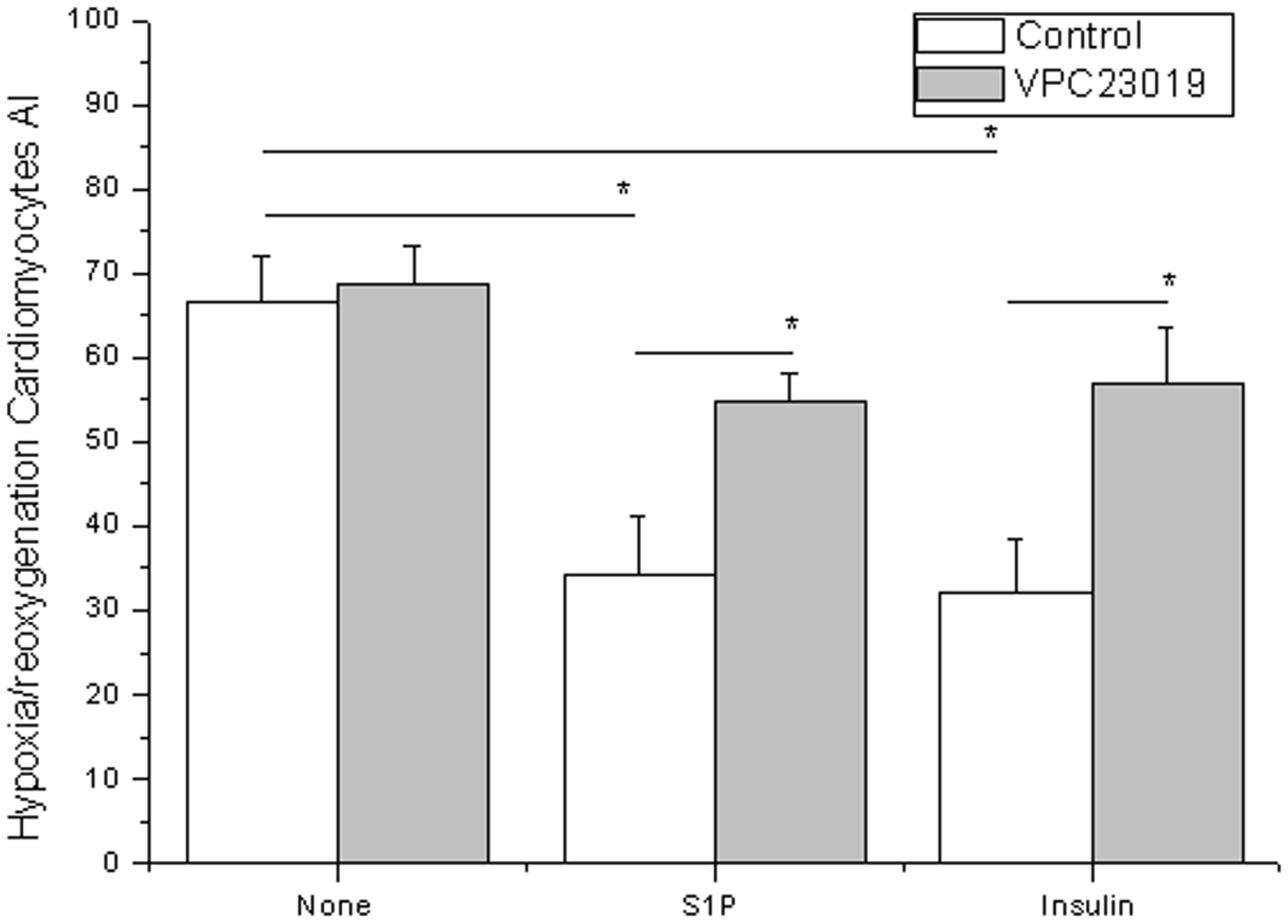
S1P receptor-mediated insulin-induced inhibition of hypoxia-reoxygenation-induced cell death in cardiomyocytes. Effects of S1P receptor antagonist on insulin in cardiomyocytes, cardiomyocytes subjected to hypoxia-reoxygenation, and cardiomyocytes undergoing reoxygenation, all either cultured with empty vehicle (vehicle, 0.05% dimethylsulfoxide) pretreated with 1 µM VPC23019, an inhibitor of S1P_1_ and S1P_3_ receptors for 30 minutes. They are shown with white and gray bars, respectively. Then cells were treated either with nothing, S1P, or insulin. Data are means ± S.E. of five independent experiments carried out in triplicate.

Insulin protected cardiomyocytes from ischemia-reperfusion-induced cell death in a dose dependent manner (Figure 4). However, treatment of cardiomyocytes with either HACPT or rSK1-siRNA abrogated the insulin-induced decrease in apoptosis (Figure 5A and B), indicating that SphK activity is important to insulin-mediated inhibition of ischemia-reperfusion-induced cell death in cardiomyocytes. In cardiomyocytes treated with S1P1 and S1P3 receptor antagonist (VPC23019), the insulin-induced decrease in apoptosis was abrogated (Figure 6), indicating the S1P receptor is involved in insulin-mediated inhibition of ischemia–reperfusion-induced cell death in cardiomyocytes. S1P served as a positive control, and the data collected here confirmed the conclusions of prior published works, that application of exogenous S1P in cultured cardiomyocytes during reoxygenation exerts prosurvival effects [15]. TUNEL staining showed that administration of S1P during reoxygenation could reduce the rate of reoxygenation-induced apoptosis, which is also a requirement for SphK 1 activity (Figure 5) and S1PR (Figure 6).

## Discussion

The cardioprotective effects of insulin were here studied through a range of cardiac preparations. Uremia is associated with enhanced susceptibility to ischemia-reperfusion injury and a loss of insulin-mediated cardioprotection [25]. Cells treated with insulin were found to be to be less vulnerable to IR-injury than control cells [26]. Insulin was found to be important factor in these cardioprotective effects. The data showed that insulin increased SphK activity in a time-dependent manner. In addition to regulating SphK activity, insulin was also responsible for rapid and transient translocation of SphK1 to the membrane fraction (Figure 1). SphK1 was found to be protective, and SphK1 overexpression was found to increase intracellular S1P content and promote cardiac cell survival [27]. The increased cell viability induced by SphK1 and subsequent S1P synthesis was caused by activation of a prosurvival pathway that included PI-3K/Akt. This pathway increased bcl-2 expression and then reduced the release of cytochrome C and caspase activation [17], [27]. SphK activation can increase intracellular S1P content, suggesting that S1P may be generated after exogenous addition of insulin. To confirm this, the rate of insulin-induced S1P generation was measured in cardiomyocytes. As expected, exogenously added insulin caused intracellular accumulation of S1P in a dose-dependent manner (Figure 2). These results strongly suggest that SphK-catalyzed formation of intracellular S1P may be relevant to the effects of insulin on cardiomyocytes.

Sphingosine-1-phosphate has been shown to be cardioprotective [15]–[17], [28]–[30]. Pre-ischemia treatment with S1P was shown to diminish the damage associated with ischemia-reperfusion injury. This protection is the result of S1P binding to specific G protein-coupled receptors, triggering a signaling cascade that leads to cardioprotection [31]. In cardiac myocytes, the S1P1 receptor subtype was the predominant subtype expressed [32].

Insulin enhanced SphK1 activity and translocation of SphK1 from the cytosol to the membrane, and the dose-dependence of the effects of insulin on S1P generation raised the issue of whether S1P generated by SphK1 was activating S1P receptors. To resolve this, FRET analyses were performed to assess S1P receptor activation. FRET efficiency was increased by insulin to the same extent as S1P stimulation of the receptor (Figure 3), confirming that S1P1 was stimulated by insulin. Treatment of cardiomyocytes with either HACPT or rSK1-siRNA abrogated the insulin-induced increase in FRET efficiency but not the increase induced by S1P (Figure 3B), indicating that the activation of S1P1 receptor by SphK1 is required. Insulin may cause S1P production. This is important for activation of the S1P1 receptor.

Cardiomyocyte apoptosis is one of the major contributors to myocardial injury after I/R. Blocking the apoptotic process was found to minimize cardiac injury induced by I/R [9], [10]. Apoptosis was found to play an important role in the pathogenesis of I/R injury. The effects of insulin on rat cardiomyocytes were investigated to determine whether insulin is involved in cardioprotection during reperfusion and whether it exerts its effects by inhibiting apoptosis. Rat cardiomyocytes were exposed to H/R alone and either treated with insulin during reperfusion or left untreated. Results showed that H/R accelerated apoptosis (data not shown). During reperfusion, insulin and S1P attenuated cardiomyocyte apoptosis. The treatment of cardiomyocytes with either HACPT or rSphK1-siRNA abrogated both insulin-induced decreases in apoptosis and the effects of S1P (Figure 5). SphK1 was found to be important to insulin- and S1P-mediated regulation of rat cardiomyocyte apoptosis. If treatment of cardiomyocytes with either S1P1 or S1P3 receptor antagonist (VPC23019) abrogated the insulin-induced decrease in apoptosis and the effects of S1P (Figure 6), then the S1P receptor was assumed to be involved in insulin- and S1P-induced inhibition of hypoxia/reoxygenation-induced cell death in cardiomyocytes. The data shown were consistent with the results of prior research indicating the cardioprotective effects of S1P requirements for SphK activity and S1PR [16], [17], [31].

Transactivation of sphingosine kinase 1/sphingosine 1-phosphate/sphingosine 1-phosphate receptor (SK/S1P/S1PR)-mediated signaling has emerged as a critical cardioprotective pathway for insulin in response to acute hypoxia/reoxygenation-injury-related apoptosis.

**Figure 7:**
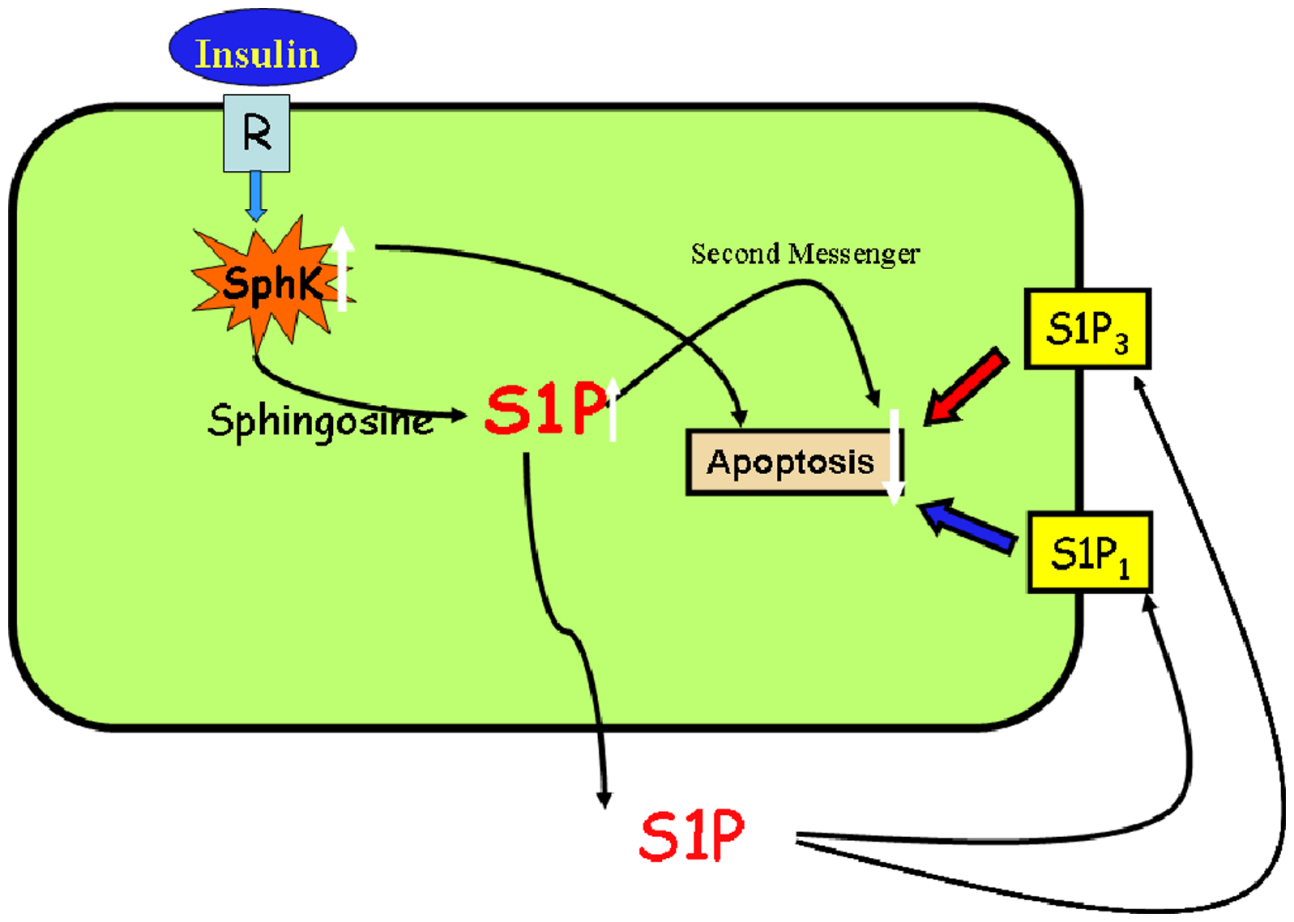
Cardioprotective pathway of insulin on acute hypoxia/reoxygenation injury-apoptosis.

Cardioprotective pathway of insulin on acute hypoxia/reoxygenation injury-apoptosis (Figure 7).
